# A machine learning approach to stratify patients with hypermobile Ehlers‐Danlos syndrome/hypermobility spectrum disorders according to disorders of gut brain interaction, comorbidities and quality of life

**DOI:** 10.1111/nmo.14957

**Published:** 2024-11-14

**Authors:** Anisa Choudhary, Asma Fikree, James K. Ruffle, Kazuya Takahashi, Olafur S. Palsson, Imran Aziz, Qasim Aziz

**Affiliations:** ^1^ Centre for Neuroscience, Surgery and Trauma, Blizard Institute, Wingate Institute of Neurogastroenterology, Barts and The London School of Medicine and Dentistry Queen Mary University of London London UK; ^2^ High Dimensional Neurology Group, UCL Queen Square Institute of Neurology University College London London UK; ^3^ Centre for Functional GI and Motility Disorders University of North Carolina at Chapel Hill Chapel Hill North Carolina USA; ^4^ Academic Department of Gastroenterology Sheffield Teaching Hospitals and University of Sheffield Sheffield UK

**Keywords:** disorders of gut brain interaction, functional dyspepsia, functional gastrointestinal disorders, hypermobile Ehlers‐Danlos syndrome, hypermobility, irritable bowel syndrome, psychological, quality of life and co‐morbidity

## Abstract

**Background:**

A high prevalence of disorders of gut‐brain interaction (DGBI) exist in patients with hypermobile Ehlers‐Danlos Syndrome (hEDS) and hypermobility spectrum disorders (HSD). However, it is unknown if clusters of hEDS/HSD patients exist which overlap with different DGBIs and whether this overlap influences presence of comorbidities and quality of life. We aimed to study these knowledge gaps.

**Methods:**

A prospectively collected hEDS/HSD cohort of 1044 individuals were studied. We undertook Uniform Manifold Approximation and Projection‐enabled (UMAP) dimension reduction to create a representation of nonlinear interactions between hEDS/HSD and DGBIs, from which individuals were stratified into clusters. Somatization, Postural Tachycardia Syndrome (PoTS), autonomic symptoms, psychological factors and quality of life were statistically compared between clusters.

**Key Results:**

The mean age of patients was 40 ± 13.2 years; 87.8% were female. Patients segregated into three clusters: Cluster 0 (*n* = 466): hEDS/HSD+ functional foregut disorders (FFD) + irritable bowel syndrome (IBS); Cluster 1 (*n* = 180): hEDS/HSD+ IBS and Cluster 2 (*n* = 337): hEDS/HSD alone. In cluster 0, we demonstrated increased somatization (*p* <0.0001), anxiety (*p* <0.0001), depression (*p* <0.0001), PoTS prevalence (*p* = 0.003), autonomic symptoms (*p* <0.0001) and reduced quality of life (*p* <0.0001) compared to cluster 2. Cluster 0 had greater comorbidity burden than cluster 1.

**Conclusions:**

Within hEDS/HSD, subgroups exist with a high prevalence of FFD and IBS. These subgroups have a higher prevalence of psychological disorders, dysautonomia and poorer quality of life compared with hEDS/HSD alone. Further research should focus on healthcare utilization, management and prognosis in hEDS/HSD and DGBI overlap.

## Graphical abstract

Within hypermobile ehlers‐danlos syndrome (hEDS)/hypermobility spectrum disorder (HSD), clusters exist with a high prevalence of functional foregut disorders and irritable bowel syndrome. These clusters have a higher prevalence of psychological disorders, dysautonomia and poorer quality of life compared with hEDS/HSD alone.

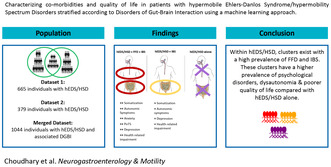

**Key points**

- Two specific DGBI clusters exist in the hEDS/HSD cohort: those with mainly functional foregut disorders (FFD) with overlapping irritable bowel syndrome (hEDS/HSD + FFD + IBS) and those with predominantly IBS (hEDS/HSD + IBS).
- Both clusters experience higher levels of somatic symptoms, health related impairment, autonomic symptoms and psychopathology compared to hEDS/HSD alone.
- Individuals with hEDS/HSD + FFD+ IBS have increased levels of anxiety disorder, autonomic symptoms and PoTS compared to both the hEDS/HSD + IBS cluster and those with hEDS/HSD alone.

## 1 INTRODUCTION

The Ehlers‐Danlos syndromes (EDS) refer to a collection of 13 inherited non‐inflammatory connective tissue disorders characterized by skin hyper‐extensibility, tissue fragility and joint hypermobility.^1^ The underlying cause may be attributed to mutations in genes encoding collagen or extracellular matrix proteins, which result in abnormalities in collagen structure and function. Currently, hypermobile Ehlers‐Danlos Syndrome (hEDS) is the only EDS subtype where both the genetic basis and pathophysiology remains unknown,^1^ hEDS is a multisystemic disorder associated with multiple comorbidities, including disorders of gut brain interaction (DGBI), postural orthostatic tachycardia syndrome (PoTS), fibromyalgia, and functional somatic syndromes.^2^ Individuals who have been previously diagnosed with some, but not all of the 2017 criteria for hEDS are currently labeled as hypermobility spectrum disorder (HSD).

Several studies have shown that hEDS/HSD patients have a higher prevalence of gastrointestinal (GI) symptoms^3^, ^4^, ^5^, ^6^ than the general population,^4^ or patients presenting to general gastroenterology clinics without hEDS/HSD.^3^ Disorders of gut‐brain interaction are particularly prevalent in hEDS/HSD, with 98% of 603 individuals found to have one DGBI, and 84% of hEDS/HSD individuals found to have two or more overlapping DGBI's; most commonly affecting the bowel, gastroduodenal, esophageal, and anorectal regions.^4^ The overlap between differing DGBIs is not uncommon, with a 36% overlap of two or more DGBIs demonstrated in general population studies.^7^

However, it is not known if clusters of hEDS/HSD and the 24 known DGBIs exist with distinct comorbidities which differentially impact physical/psychological health and quality of life. Identification of such specific disease clusters may have implications for understanding pathophysiology and for improving management of these complex disorders.

Given that both DGBI's and hEDS/HSD are by definition complex disorders governed by multiple interacting factors, any overlap is plausibly best characterized with mathematical modeling techniques such as machine learning capable of comprehending high‐dimensional and nonlinear features. Thus, we aimed to determine if (i) Within patients with hEDS/HSD, distinct clusters exist with a high prevalence of specific DGBI overlap, and (ii) determine differences in non‐GI comorbidities and quality of life between clusters. We hypothesize that those with the largest number of DGBIs will have more non‐GI comorbidities and lower quality of life in comparison with those with no DGBI overlap.

## 2 MATERIALS AND METHODS

### 2.1 Study population

In this study, hEDS refers to individuals satisfying the 2017 international classification of hypermobile Ehlers‐Danlos Syndrome^8^ and HSD refers to patients with previous diagnoses of Ehlers‐Danlos Syndrome type 3 (EDS III), Ehlers‐Danlos Syndrome Hypermobility type (EDS‐HT) or Joint Hypermobility Syndrome (JHS) historically made by the Brighton or Villefranche Criteria and have not been since reassessed using the 2017 criteria^9^; or to those patients diagnosed since 2017 who have some but not all features of hEDS.

Two existing databases of hEDS/HSD patients were used in this study.^3^, ^4^

*Dataset 1* (D1): individuals were recruited from the patient support organization EDS UK in October 2018 and outcomes from this data have previously been reported.^4^ The dataset included information on age, sex, hEDS/HSD and PoTS diagnosis (previously made by a medical professional), DGBI diagnoses (ROME IV), non‐GI related somatization [Patient Health Questionnaire‐12 (PHQ‐12)],^4^, ^10^ quality of life [Short Form Survey 8 (SF‐8)],^11^ anxiety symptoms [General anxiety disorder questionnaire (GAD‐7)]^12^ and depression symptoms [Patient health questionnaire‐9 (PHQ‐9)].^13^ Details and interpretations of the questionnaires used can be found in Supplementary document 1.

*Dataset* 2 (D2): consisted of individuals recruited from a combination of: secondary and tertiary care gastroenterology clinics, and from primary care settings between April 2010 and April 2012. As these patients were assessed before 2017, they were assessed using the previous Brighton criteria^9^ (by AF). Consequently, the patients in this dataset would meet the current 2017 criteria for HSD, however, as we could not exclude that some of them would also meet the 2017 criteria for hEDS if tested again, we called them hEDS/HSD. The dataset included information on age, sex, HSD diagnosis, DGBI diagnoses (ROME III), non‐GI related somatization (PHQ‐12),^4^, ^10^ quality of life [Short Form Survey 36 (SF‐36)],^14^ anxiety and depression symptoms [The Symptom Checklist—90 (SCL‐90)]^15^ and autonomic symptoms (COMPASS questionnaire).^16^ Details and interpretations of the questionnaires used can be found in Supplementary Document 1.

*Merged dataset*: Data on DGBI diagnosis and hEDS/HSD diagnosis from all existing individuals from D1 and D2 were merged to form one combined dataset. This culminated in a large geographically varied sample size acquired from across the United Kingdom, deemed preferrable as a measure to boost generalizability of any findings. The disadvantages of this method included the different populations and the different methods of assessment within the two cohorts. These disadvantages were addressed and are described in the section below.

### 2.2 Machine learning

Python 3.8, a programming language and scikit learn, an open‐source statistical library were used for all machine learning (ML) processes described within this manuscript.^17^

#### 2.2.1 Identification of dataset as a confounding factor

As patient data in the merged dataset was compiled via two different geographical sources using different methodologies and criteria to assess for the presence of DGBI and hEDS/HSD, we fitted a logistic regression to determine if a model could predict which dataset patients belonged to according to their DGBI status and hEDS/HSD diagnosis. If such a model achieved only chance accuracy, it would suggest the pooling of the datasets an appropriate approach, whereas a model that could delineate the dataset would suggest confounding factors exist in this approach. The DGBIs used in this article are summarized under the heading “included in cluster analysis” in Table 1. In building this quality control model, 70% of patients were randomly allocated for the training of our model, reserving the remaining 30% for model testing. The performance of the model was tested by calculation of accuracy, precision, recall, *f*‐value, receiver operating characteristic curve (ROC) and area under the ROC curve (AUC). Furthermore, we used the chi‐square test to compare sex distribution and DGBI prevalence between the two datasets, as well as a t‐test to compare age.

**TABLE 1:** The presence of DGBI in: Dataset 1 (hEDS/HSD, *n* = 665); dataset 2 (hEDS/HSD, *n* = 379); merged Dataset and included in the final dataset (hEDS/HSD, *n* = 1044). (%).

DGBI	Dataset 1 (ROME IV) *n*, (%)	Dataset 2 (ROME III) *n*, (%)	Merged Dataset *n*, (%)	Included in cluster analysis *n*, (%)
IBS	✔ 371 (55.8)	✔ 124 (32.7)	✔ 495 (47.4)	✔ 495 (47.4)
IBS‐C	✔ 129 (19.4)	✔ 34 (9)	✔ 163 (15.6)	✘
IBS‐D	✔ 81 (12.2)	✔ 27 (7.1)	✔ 108 (10.3)	✘
IBS‐M	✔ 156 (23.5)	✔ 59 (15.6)	✔ 215 (20.6)	✘
IBS‐U	✔ 5 (0.8)	✔ 4 (1.1)	✔9 (0.9)	✘
Functional dyspepsia	✔ 382 (57.4)	✔ 113 (29.8)^a^	✔ 495 (47.4)	✔ 495 (47.4)
Postprandial distress syndrome	✔ 332 (49.9)	✔ 74 (19.5)	✔ 406 (38.9)	✔ 406 (38.9)
Epigastric pain syndrome	✔ 221 (33.2)	✔ 11 (2.9)	✔ 232 (22.2)	✔ 232 (22.2)
Dysphagia	✔ 282 (42.4)	✔ 24 (6.3)	✔ 306 (29.3)	✔ 306 (29.3)
Proctalgia fugax	✔195 (29.3)	✔ 29 (7.7)	✔ 224 (21.4)	✔ 224 (21.4)
Rumination	✔ 198 (29.8)	✔ (0)	✔ 198 (18.9)	✔ 198 (18.9)
Functional heartburn	✔ 123 (18.5)	✔ 3 (0.8)	✔ 126 (12.1)	✔ 126 (12.1)
Fecal incontinence	✔ 118 (17.7)	✔ 1 (0.3)	✔ 119 (11.4)	✔ 119 (11.4)
Chronic nausea and vomiting	✔ 102 (15.3)	✔ 6 (1.6)	✔ 108 (10.3)	✔ 108 (10.3)
Functional chest pain	✔ 84 (12.6)	✔ 16 (4.2)	✔ 100 (9.6)	✔ 100 (9.6)
Functional constipation	✔ 81 (12.2)	✔ 5 (1.3)	✔ 86 (8.2)	✔ 86 (8.2)
Belching	✔ 77 (11.6)	✔ 2 (0.5)	✔ 79 (7.6)	✔ 79 (7.6)
Cyclic vomiting	✔ 66 (9.9)	✔ (0)	✔ 66 (6.3)	✔ 66 (6.3)
Unspecified functional bowel disorder	✔ 62 (9.3)	✔ 8 (2.1)	✔ 70 (6.7)	✔ 70 (6.7)
Functional bloating	✔ 18 (2.7)	✔ (0)	✔ 18 (1.7)	✔ 18 (1.7)
Reflux hypersensitivity	✔ (15.6)	✘	✘	✘
Globus	✔ 11 (1.7)	✔ 12 (3.2)	✔ 23 (2.2)	✔ 23 (2.2)
Central abdominal pain syndrome	✔ 3 (0.5)	✔ 3 (0.8)	✔ 6 (0.6)	✔ 6 (0.6)
Functional biliary pain	✔ (1.5)	✘	✘	✘
Cannabinoid hyperemesis	✔ (0.3)	✘	✘	✘
Functional DIARRHEA	✔ 34 (5.1)	✔ 10 (2.6)	✔ 44 (4.2)	✔ 44 (4.2)
Levator ani syndrome	✔ (18.9)	✘	✘	✘
Opioid induced constipation	✔ (9.2)	✘	✘	✘

^a^ Twenty‐eight patients had missing data on postprandial distress syndrome and epigastric pain syndrome status.

#### 2.2.2 Uniform Manifold Approximation and Projection enhanced clustering

Uniform Manifold Approximation and Projection (UMAP), a computational, non‐linear, dimension reduction technique was used as a steppingstone to identify clusters of patients from the high‐dimensional clinical comorbidity data (Table 1).^18^, ^19^ This approach was akin to UMAP‐enhanced clustering, wherein after reducing data to a two‐dimensional feature space, the features were passed through an unsupervised density‐based clustering algorithm; HDBSCAN.^20^

### 2.3 Downstream conventional statistical analysis

Having identified clusters of hEDS/HSD individuals determined from DGBI comorbidities, we statistically tested for differences in somatization, health related quality of life (HrQol), anxiety, depression, autonomic symptoms via COMPASS questionnaire, PoTS prevalence and Beighton scores (which serve as a marker of joint hypermobility) between the clusters. However, as both datasets 1 and 2 had used different questionnaires to assess for these parameters, the clusters formed using UMAP and HDBSCAN were separated back into their original datasets prior to downstream statistical analysis (D1 and D2).

### 2.4 Statistical analysis

All data analysis was conducted using SPSS Statistics 26. Continuous data was summarized using median and IQR.

The Shapiro–Wilks test was used to test for normality and subsequently a one‐way non‐parametric ANOVA (Kruskal–Wallis test) was used for all non‐normally distributed data. A value of *p* <0.05 was considered statistically significant for comparisons. Significance values were adjusted by the Bonferroni correction for multiple tests.

## 3 RESULTS

### 3.1 Study population (Table 1)

Dataset 1 consisted of 665 individuals, with hEDS/HSD. 96% (641) of these were female, and 4%^22^ were male. The mean age was 39 ± 12.8 years; age range of 18–75 years. The DGBI included in dataset 1 can be found in Table 1.

Dataset 2 consisted of 379 individuals with HSD of which 73% (276) were female. The mean age was 41 ± 6.8 years with an age range of 18–70 years. The DGBI included in dataset 2 can be found in Table 1. The proportion of patients, demographics and DGBI prevalence from primary versus secondary and tertiary care in dataset 2 can be found in Tables S1 and S2.

The combined dataset consisted of 1044 individuals with hEDS/HSD of which 87.8% (917) were female. The mean age of patients was 40 ± 13.2 years; age range 18–75 years. The DGBI included in dataset 1, dataset 2 and the merged dataset can be found in Table 1.

### 3.2 Machine learning

#### 3.2.1 Determining con‐founding factors within the combined dataset

Whilst significant—univariate–differences in sex, age and DGBI prevalence were observed between the two datasets (Tables S3 and S4), a multivariate–logistic regression model seeking to predict the dataset from which patients came from performed entirely by chance.

**FIGURE 1:**
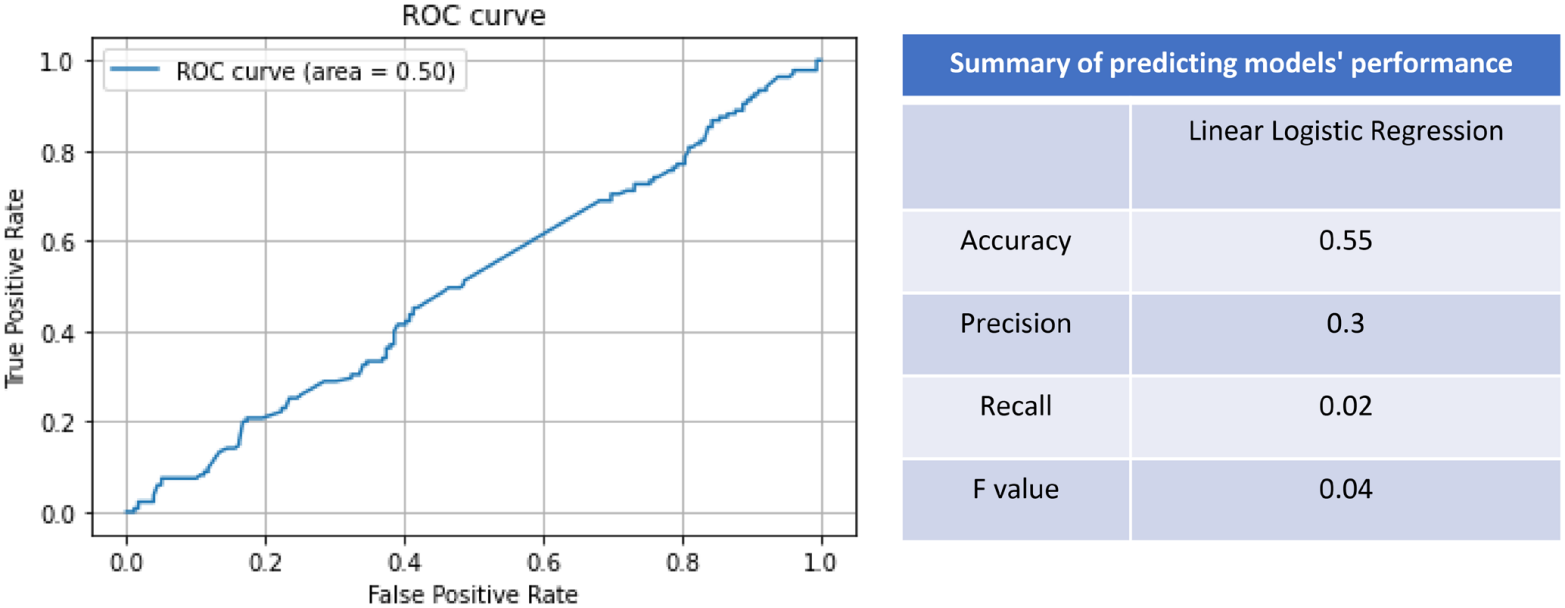
A linear logistic regression model demonstrating that the site of recruitment does not act as a confounding factor.

That is the model could not detect which dataset the patients belonged to based on DGBI and hEDS/HSD status. Consequently, we concluded that the dataset from which patients were derived did not confound the data and therefore pooled the datasets to curate the larger sample size for subsequent cluster analyses. The performance of the linear logistic regression model is summarized in Figure 1 (AUC: 0.50, accuracy: 0.55, precision: 0.3, recall: 0.02, *f‐*value: 0.04).

#### 3.2.2 Uniform Manifold Approximation and Projection enhanced clustering

**FIGURE 2:**
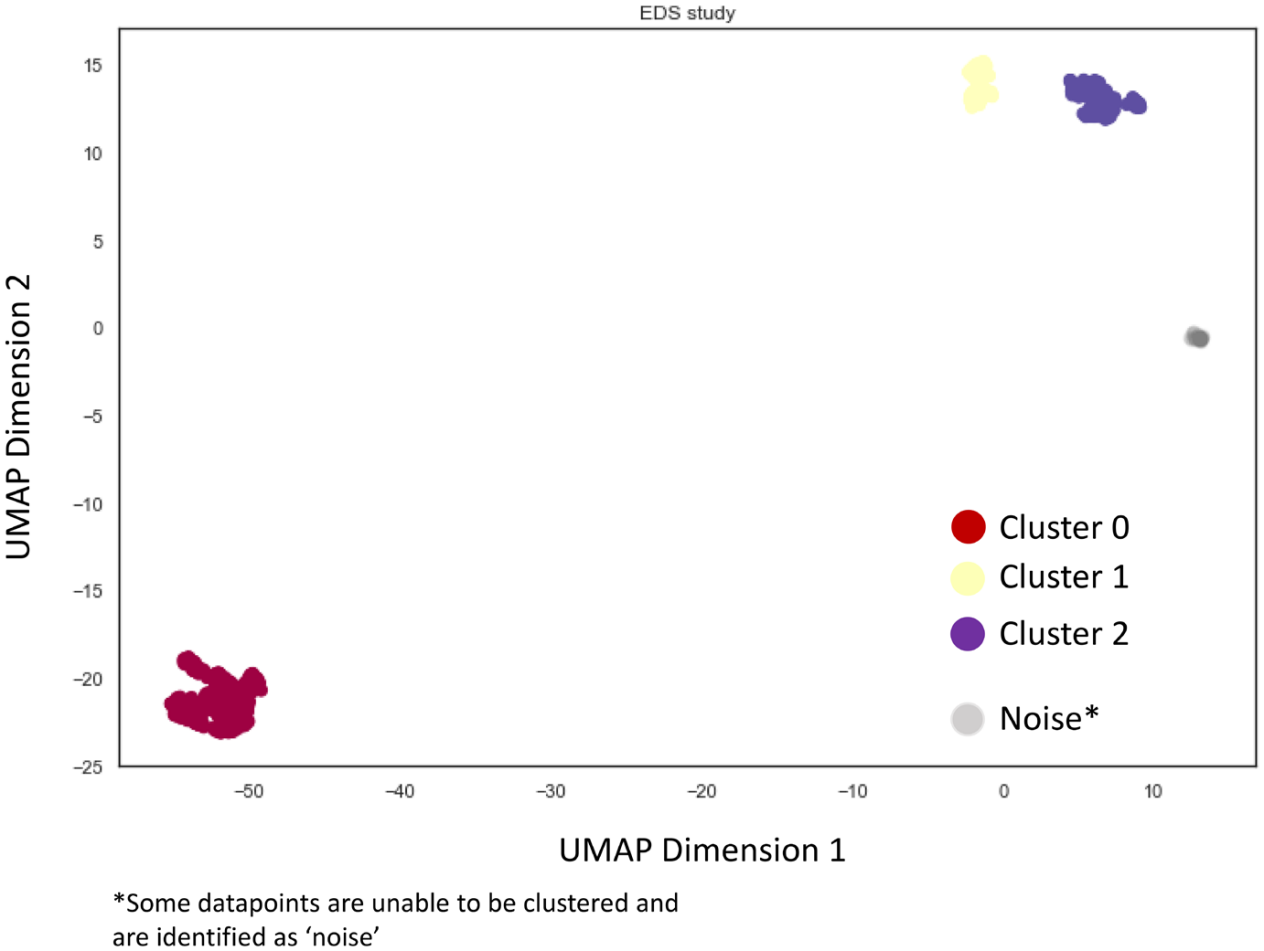
Clustering by unsupervised UMAP combined with HDBSCAN, demonstrating the formation of 3 clusters.

Application of UMAP and HDBSCAN segregated 983 individuals (out of our 1044) into three distinct clusters, the demographics of which can be found in Table 2. The 983 Individuals were clustered based on their hEDS/HSD status and DGBI diagnosis (Figure 2 and Table 3). The remaining 61 could not be assigned to a cluster and were classified as ‘outliers’ and were discarded from downstream analyses (represented by gray dots in Figure 2). The characteristics of the 61 individuals classified as ‘noise’, all of whom had an unspecified functional bowel disorder can be found in Tables S5 and S6. Individuals appeared to cluster contingent on the presence of hEDS/HSD and accumulating burden of comorbid DGBIs. Cluster 0 (*n* = 466) contained a set of patients with hEDS/HSD (100%) and functional foregut disorders (FFD). The FFD included functional dyspepsia (100%), dysphagia (49.4%), functional heartburn (21.9%), belching (14.2%), chronic nausea and vomiting (18.7%) and rumination (30.3%). The FFD were significantly increased in cluster 0 compared to the other two clusters as represented in Table 3 (*p* ≤ 0.001). Additionally, 67.6% of patients in this cluster suffered from IBS. Consequently, this cluster was labeled as hEDS/HSD + FFD + IBS.

**TABLE 2:** Demographics of the three clusters formed.

Clinical setting (*n*)	Age range (years)	Sex (*n*)
Cluster 0	18–75	Female (445) Male (21)
Cluster 1	19–72	Female (167) Male (13)
Cluster 2	18–75	Female (250) Male (87)

**TABLE 3:** Summary of hEDS/HSD and DGBI present in each of the three clusters.

Variable (hEDS/HSD diagnosis and DGBI)	Cluster 0 *n* = 466 (*n*, %) hEDS/HSD + FFD + IBS	Cluster 1 *n* = 180 (*n*, %) hEDS/HSD + IBS	Cluster 2 *n* = 337 (*n*, %) hEDS/HSD alone	*p*‐value (Cluster 0 vs. Cluster 2)	*p*‐value (Cluster 1 vs. Cluster 2)	*p*‐value (Cluster 0 vs. Cluster 1)
hEDS/HSD	466 (100)	180 (100)	337 (100)	^a^	^a^	^a^
Functional dyspepsia	466 (100)	18 (10)	10 (3)	***<0.0001***	0.383	***<0.0001***
Postprandial distress syndrome	405 (86.9)	1 (0.6)	0			
Epigastric pain syndrome	232 (49.8)	0 (0)	0			
Irritable bowel syndrome	315 (67.6)	180 (100)	0	***<0.0001***	***<0.0001***	***<0.0001***
Functional heartburn	102 (21.9)	11 (6.1)	9 (2.7)	***<0.0001***	0.160	***<0.0001***
Functional chest pain	55 (11.8)	26 (14.4)	16 (4.7)	***0.002***	***<0.0001***	1.000
Globus	15 (3.2)	4 (2.2)	3 (0.9)	^a^	^a^	^a^
Dysphagia	230 (49.4)	39 (21.7)	28 (8.3)	***<0.0001***	***<0.0001***	***<0.0001***
Belching	66 (14.2)	7 (3.9)	3 (0.9)	***<0.0001***	0.055	***0.001***
Rumination	141 (30.3)	27 (15)	20 (5.9)	***<0.0001***	***0.002***	***<0.0001***
Chronic nausea and vomiting syndrome	87 (18.7)	13 (7.2)	7 (2.1)	***<0.0001***	0.012	***0.001***
Cyclical vomiting	54 (11.6)	6 (3.3)	6 (1.8)	***<0.0001***	0.792	0.004
Functional constipation	47 (10.1)	0	39 (11.6)	1.0000	***<0.0001***	***<0.0001***
Functional diarrhea	12 (2.6)	0	32 (9.5)	***<0.0001***	***<0.0001***	0.089
Functional bloating	1 (0.2)	0	17 (5)	***<0.0001***	0.007	1.000
Unspecified functional bowel disorder	9 (1.9)	0	0	***0.031***	^b^	0.181
Central abdominal pain syndrome	1 (0.2)	0	4 (1.2)	^c^	^c^	^c^
Fecal incontinence	96 (20.6)	9 (5)	10 (3)	***<0.0001***	0.726	**<0.0001**
Proctalgia fugax	139 (29.8)	45 (25)	29 (8.6)	***<0.0001***	<0.0001	0.669

^a^ Multiple comparisons are not performed because the overall test does not show significant differences across samples.

^b^ Unable to compute as all sample medians in this pair are less than or equal to the hypothesised median.

^c^ Multiple comparisons are not performed because the overall test does not show significant differences across samples.

Cluster 1 (*n* = 180) contained a group of patients with hEDS/HSD (100%) and IBS (100%) and had significantly less FFD than cluster 0 [functional dyspepsia (10% vs. 100%), dysphagia (21.7% vs. 49.4%) functional heartburn (6.1% vs. 21.9%) belching (3.9% vs. 14.2%), chronic nausea and vomiting (7.2% vs. 18.7%) and rumination (15% vs. 30.3%), (*p* ≤ 0.001)]. Consequently, this cluster was labeled hEDS/HSD + IBS.

Lastly, cluster 2 (*n* = 337) consisted of patients with hEDS/HSD (100%) and very few DGBI, with the highest prevalence being that of functional constipation existing in 11.6% of patients. Presence of functional foregut disorders were significantly less than cluster 0 [functional dyspepsia (3% vs. 100%), dysphagia (8.3% vs. 49.4%) functional heartburn (2.7% vs. 21.9%) belching (0.9% vs. 14.2%), chronic nausea and vomiting (2.1% vs. 18.7%) and rumination (5.9% vs. 30.3%), (*p* ≤ 0.0001)] and the prevalence of IBS was 0%. Consequently, this cluster was labeled as hEDS/HSD alone.

**FIGURE 3:**
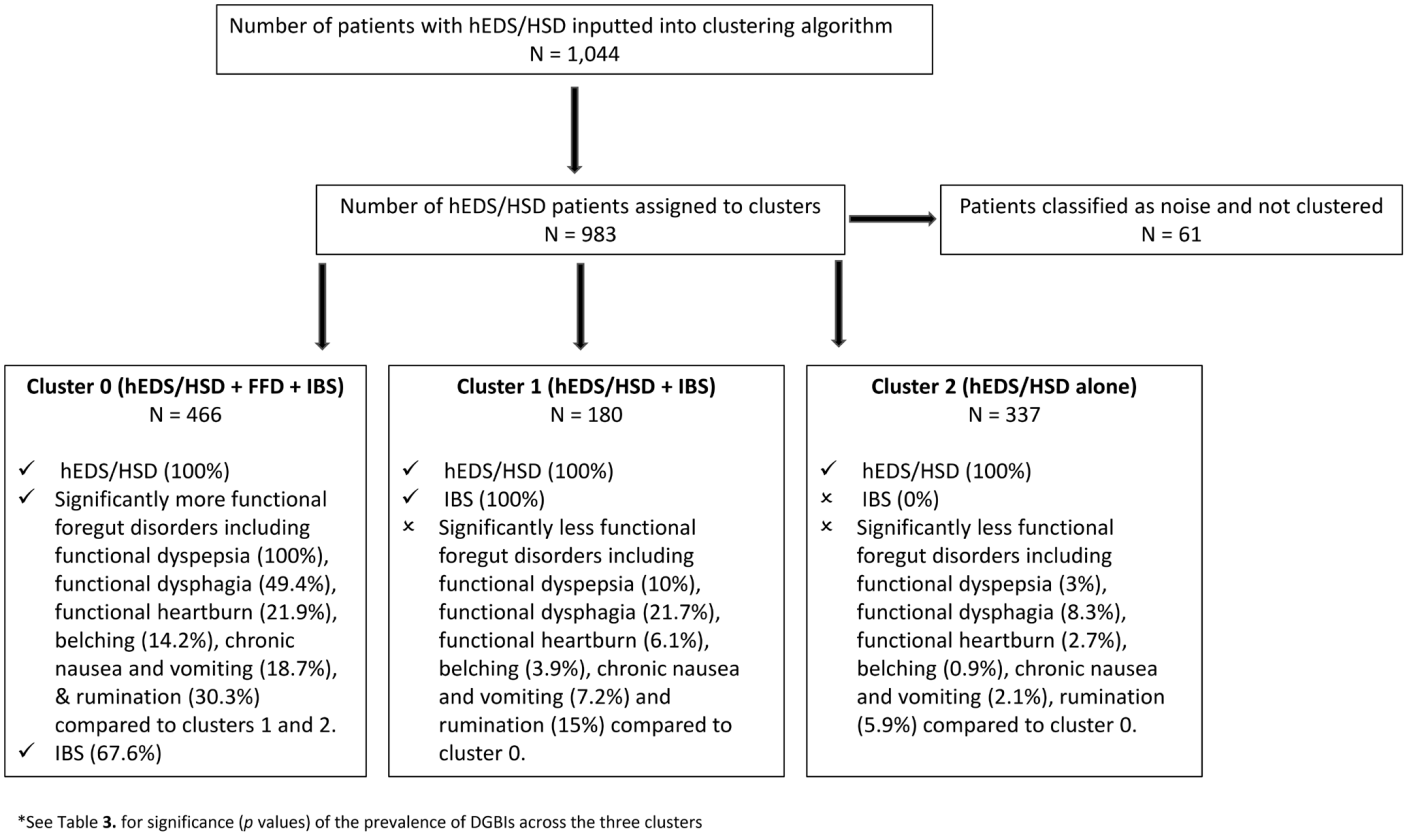
Illustration of cluster allocations.

A flow diagram to illustrate cluster allocations can be found in Figure 3 and a summary of the proportion of patients in each cluster with hEDS/HSD and DGBI diagnoses with correlating *p*‐values can be found in Table 3.

### 3.3 Downstream statistical analysis

Clusters derived from the merged dataset (Table 4) were split back into D1 (Table 5) and D2 (Table 6) for downstream statistical analysis.

**TABLE 4:** Clusters derived from the merged dataset, split back into D1 and D2.^b^

Cluster	Dataset 1 (D1) *n* = 612 *n*, (%)	Dataset 2 (D2) *n* = 371 *n*, (%)
0 (hEDS/HSD + FFD + IBS)	382 (62.4)^a^	84 (22.6)^a^
1 (hEDS/HSD + IBS)	114 (18.6)	66 (17.8)
2 (hEDS/HSD alone)	116 (19)	221 (59.6)

^a^ The differences in % in the hEDS/HSD + FFD + IBS cluster between D1 and D2 may be attributed to the composition of the patient groups; whilst D1 may predominantly include patients with hEDS, D2 compromises of patients with HSD.

^b^ Sixty‐one patients were not assigned to clusters as they were detected as ‘outliers.’ These are represented by grey dots in figure 2. Characteristics of these 61 individuals can be found in supplementary tables 5 and 6.

**TABLE 5:** Presence of bothersome non‐GI somatic symptoms over the past 4 weeks; quality of life scores; depression Scores; anxiety Scores and PoTs status in: HEDS/HSD + FFD + IBS vs. hEDS/HSD alone; hEDS/HSD + IBS vs. hEDS/HSD alone; hEDS/HSD + FFD + IBS vs. hEDS + IBS in dataset 1 (D1).

Variable	Cluster 0 hEDS/HSD + FFD + IBS (*n* = 382)	Cluster 2 hEDS/HSD alone (*n* = 116)	*p* value	Cluster 1 hEDS/HSD + IBS (*n* = 114)	Cluster 2 hEDS/HSD alone (*n* = 116)	*p*‐value	Cluster 0 hEDS/HSD + FFD + IBS (*n* = 382)	Cluster 1 hEDS/HSD + IBS (*n* = 114)	*p‐*value
PHQ‐12 scores									
PHQ‐12 total Median, [IQR]	16 [13–18]	11.5 [9–14]	(***<0.0001***)	13.5 [11.8–15]	11.5 [9–14]	(***0.005***)	16 [13–18]	13.5 [11.8–15]	(***<0.0001***)
Back Pain (%)	68	54.3	(***0.01***)	75.4	54.3	(***0.002***)	68	75.4	(1.00)
Pain in arms/legs/joints (%)	87.2	73.3	(***0.001***)	87.7	73.3	(***0.01***)	87.2	87.7	(0.632)
Menstrual cramps (%)	35.3	24.1	(***0.034***)	32.5	24.1	(0.895)	35.3	32.5	(1.000)
Headaches (%)	45	18.1	(***<0.0001***)	34.2	18.1	(***0.022***)	45	34.2	(0.062)
Chest pain (%)	18.8	6	(***<0.0001***)	7.9	6	(1.000)	18.8	7.9	(***0.003***)
Dizziness (%)	49	23.3	(***<0.0001***)	29.8	23.3	(0.232)	49	29.8	(***0.001***)
Fainting spells (%)	13.3	3.4	(***<0.0001***)	2.6	3.4	(1.000)	13.3	2.6	(***<0.0001***)
Palpitations (%)	49	16.4	(***<0.0001***)	28.1	16.4	(0.098)	49	28.1	(***<0.0001***)
SOB (%)	31.2	16.4	(***<0.0001***)	18.4	16.4	(1.000)	31.2	18.4	(***0.003***)
Dyspareunia (%)	23.3	9.5	(***0.002***)	16.7	9.5	(0.636)	23.3	16.7	(0.635)
Lethargy (%)	91.4	81.9	(***0.019***)	90.4	81.9	(0.209)	91.4	90.4	(1.000)
Insomnia (%)	70.4	54.3	(***0.019***)	60.5	54.3	(1.000)	70.4	60.5	(0.843)
Quality of Life Scores, Median [IQR]									
Physical Func. (PF)	31.46 [30.3–40.1]	36.16 [30.3–40.1]	(***<0.0001***)	34.81 [30.3–40.1]	36.16 [30.3–40.1]	(1.000)	31.46 [30.3–40.1]	34.81 [30.3–40.1]	(***<0.0001***)
Role physical (RP)	29.06 [23–28.3]	35.14 [28.3–38.7]	(***<0.0001***)	32.69 [28.3–38.7]	35.14 [28.3–38.7]	(0.566)	29.06 [23–28.3]	32.69 [28.3–38.7]	(***<0.0001***)
Bodily Pain (BP)	33.29 [31.5–40.1]	37.27 [31.5–40.1]	(***<0.0001***)	35.14 [31.5–40.1]	37.27 [31.5–40.1]	(0.238)	33.29 [31.5–40.1]	35.14 [31.5–40.1]	(***0.023***)
General health (GH)	33.4 [32.6–38.4]	38.07 [32.6–44.4]	(***<0.0001***)	36.14 [32.6–38.4]	38.07 [32.6–44.4]	(0.387)	33.4 [32.6–38.4]	36.14 [32.6–38.4]	(***0.001***)
Vitality (VT)	37.35 [35.8–45.1]	40.65 [35.8–45.2]	(***<0.0001***)	38.77 [35.8–45.2]	40.65 [35.8–45.2]	(0.396)	37.35 [35.8–45.1]	38.77 [35.8–45.2]	(0.159)
Social func. (SF)	33.14 [29.5–40.4]	38.62 [29.5–49.5]	(***<0.0001***)	35.90 [29.5–40.4]	38.62 [29.5–49.5]	(0.287)	33.14 [29.5–40.4]	35.90 [29.5–40.4]	(***0.010***)
Role emotional (RE)	38.61 [29.3–45.7]	42.92 [38.1–52.4]	(***<0.0001***)	41.87 [38.1–45.7]	42.92 [38.1–52.4]	(1.000)	38.61 [29.3–45.7]	41.87 [38.1–45.7]	(***0.014***)
Mental health (MH)	37.62 [31.6–49.6]	41.85 [31.6–49.6]	(***0.004***)	39.15 [31.6–49.6]	41.85 [31.6–49.6]	(0.363)	37.62 [31.6–49.6]	39.15 [31.6–49.6]	(1.000)
Depression, Anxiety, PoTs status, Median [IQR]									
Depression score (PHQ9)	15.76 [11–22]	10.73 [6–14]	(***<0.0001***)	13.03 [9–17]	10.73 [6–14]	(***0.035***)	15.76 [11–22]	13.03 [9–17]	(***0.001***)
Anxiety Score (GAD7)	10.71 [5–16]	7.39 [3–11]	(***<0.0001***)	9.92 [4–13.3]	7.39 [3–11]	(0.418)	10.71 [5–16]	9.92 [4–13.3]	(***0.033***)
PoTs frequency (%)	45.5	27.6	(***0.003***)	31.6	27.6	(1.000)	45.5	31.6	(***0.043***)

*Note*: Significance values have been adjusted by the Bonferroni correction for multiple tests. Statistically significant values were mentioned in bold and italics.

**TABLE 6:** Presence of bothersome non‐GI somatic symptoms over the past 4 weeks; quality of life scores; depression Scores; anxiety Scores, Beighton status and COMPASS scores in: HEDS/HSD + FFD + IBS versus hEDS/HSD alone; hEDS/HSD + IBS vs. hEDS/HSD alone; hEDS/HSD + FFD + IBS versus hEDS + IBS in dataset 2 (D2).

Variable	Cluster 0 hEDS/HSD + FFD + IBS (*n* = 84)	Cluster 2 hEDS/HSD alone (*n* = 221)	*p*‐value	Cluster 1 hEDS/HSD + IBS (*n* = 66)	Cluster 2 hEDS/HSD alone (*n* = 221)	*p*‐value	Cluster 0 hEDS/HSD + FFD + IBS (*n* = 84)	Cluster 1 hEDS/HSD + IBS (*n* = 66)	*p*‐value
PHQ‐12 scores									
PHQ‐12 Total Median, [IQR]	12 [10–15.8]	5 [0–8]	(***<0.0001***)	10 [7–14]	5 [0–8]	(***<0.0001***)	12 [10–15.8]	10 [7–14]	(0.177)
Back Pain (%)	58.3	21.6	(***<0.0001***)	54.5	21.6	(***<0.0001***)	58.3	54.5	(1.000)
Pain in arms/legs/joints (%)	65.1	24.3	(***<0.0001***)	54.5	24.3	(***<0.0001***)	65.1	54.5	(1.000)
Menstrual cramps (%)	21.7	9	(***<0.0001***)	14.3	9	(0.063)	21.7	14.3	(1.000)
Headaches (%)	28.6	8.4	(***<0.0001***)	54.5	8.4	(***<0.0001***)	28.6	54.5	(0.674)
Chest Pain (%)	25	5.6	(***<0.0001***)	12.1	5.6	(0.148)	25	12.1	(***0.024***)
Dizziness (%)	39.3	21.4	(***<0.0001***)	21.2	21.4	(***<0.0001***)	39.3	21.2	(***0.029***)
Fainting spells (%)	12	2.3	(***<0.0001***)	3	2.3	(***0.036***)	12	3	(***0.039***)
Palpitations (%)	22.9	5.6	(***<0.0001***)	19.7	5.6	(***<0.0001***)	22.9	19.7	(1.000)
SOB (%)	24.1	4.2	(***<0.0001***)	15.4	4.2	(***0.003***)	24.1	15.4	(0.228)
Dyspareunia (%)	15.2	3.8	(***0.016***)	11.5	3.8	(0.469)	15.2	11.5	(1.000)
Lethargy (%)	77.4	27.9	(***<0.0001***)	68.2	27.9	(***<0.0001***)	77.4	68.2	(1.000)
Insomnia (%)	56	22.8	(***<0.0001***)	40.9	22.8	(***<0.0001***)	56	40.9	(1.000)
Quality of Life Scores, Median [IQR]									
Physical Func. (PF)	41.4 [15–75]	71.6 [50–95]	(***<0.0001***)	55.8 [20–82.3]	71.6 [50–95]	(***0.007***)	41.4 [15–75]	55.8 [20–82.3]	(0.171)
Role Physical (RP)	21.3 [0–43.8]	52.7 [0–100]	(***<0.0001***)	33.5 [0–75]	52.7 [0–100]	(***0.043***)	21.3 [0–43.8]	33.5 [0–75]	(0.305)
Bodily Pain (BP)	23 [10–32]	52.2 [24–70]	(***<0.0001***)	37.2 [22–57]	52.2 [24–70]	(***0.005***)	23 [10–32]	37.2 [22–57]	(***0.018***)
General health (GH)	27.6 [10–40]	46.1 [25–65]	(***<0.0001***)	35.9 [15–51.3]	46.1 [25–65]	(***0.034***)	27.6 [10–40]	35.9 [15–51.3]	(0.321)
Vitality (VT)	30.2 [10–50]	42.7 [25–60]	(***0.001***)	34.8 [20–55]	42.7 [25–60]	(0.140)	30.2 [10–50]	34.8 [20–55]	(1.000)
Social func. (SF)	33.9 [12–50]	60.7 [37–87]	(***<0.0001***)	44.7 [25–75]	60.7 [37–87]	(***0.004***)	33.9 [12–50]	44.7 [25–75]	(0.304)
Role emotional (RE)	47.7 [0–100]	62.3 [0–100]	(***0.045***)	65 [33–100]	62.3 [0–100]	(1.000)	47.7 [0–100]	65 [33–100]	(0.091)
Mental health (MH)	60 [45–76]	62.6 [48–80]	^a^	62.6 [48–80]	62.6 [48–80]	^a^	60 [45–76]	62.6 [48–80]	7
Depression, Anxiety, PoTs status, Median [IQR]									
Depression score (scl‐90)	1.5 [0.7–2.3]	0.9 [0–1.3]	(***<0.0001***)	1.1[0.3–1.8]	0.9 [0–1.3]	(***0.021***)	1.5 [0.7–2.3]	1.1[0.3–1.8]	(0.087)
Anxiety Score (scl‐90)	1.2 [0.5–1.9]	0.6 [0–0.8]	(***<0.0001***)	0.8 [0.2–1.1]	0.6 [0–0.8]	(*0.055*)	1.2 [0.5–1.9]	0.8 [0.2–1.1]	(***0.05***)
Beighton (Mean)	4 [2–6]	3.3 [2–5]	^a^	3.6 [2–5]	3.3 [2–5]	^a^	4 [2–6]	3.6 [2–5]	7
Autonomic Symptom Profile (COMPASS scores), Median, [IQR]									
Urinary	20 [10–40]	0 [0–20]	(***<0.0001***)	20 [0–42.5]	0 [0–20]	(***<0.0001***)	20 [10–40]	20 [0–42.5]	(1.000)
Diarrhea	40 [0–60]	0 [0–40]	(***<0.0001***)	40 [0–60]	0 [0–40]	(***<0.0001***)	40 [0–60]	40 [0–60]	(***0.023***)
Gastroparesis	50 [33.3–66.7]	0 [0–33.3]	(***<0.0001***)	33.33 [16.7–50]	0 [0–33.3]	(***<0.0001***)	50 [33.3–66.7]	33.33 [16.7–50]	(***<0.0001***)
Orthos tatic intolerance	65.63 [37.5–81.3]	6.25 [0–43.8]	(***<0.0001***)	43.75 [12.5–64]	6.25 [0–43.8]	(***<0.0001***)	65.63 [37.5–81.3]	43.75 [12.5–64]	(***0.003***)
Reflex Syncope	0 [0–15]	0 [0–0]	(***0.009***)	0 [0–0]	0 [0–0]	(0.233)	0 [0–15]	0 [0–0]	(1.000)
Vasomotor	37.5 [0–62.5]	0 [0–0]	(***<0.0001***)	0 [0–56.3]	0 [0–0]	(***<0.0001***)	37.5 [0–62.5]	0 [0–56.3]	(1.000)
Constipation	28.5 [0–71.4]	0 [0–14.8]	(***<0.0001***)	28.57 [14.3–46.2]	0 [0–14.8]	(***<0.0001***)	28.5 [0–71.4]	28.57 [14.3–46.2]	(1.000)

*Note*: Significance values have been adjusted by the Bonferroni correction for multiple tests. Statistically significant values were mentioned in bold and italics.

^a^ Multiple comparisons are not performed because the overall test does not show significant differences across samples.

#### 3.3.1 Somatization—PHQ‐12 scores (Tables 5 and 6)

Somatization scores were highest in patients with hEDS/HSD + FFD + IBS. This cluster had significantly higher median PHQ‐12 scores (*p* ≤ 0.0001) and significantly more somatization in all domains (*p* ≤ 0.05) compared to hEDS/HSD alone. Additionally, within the somatic symptoms, this cohort had significantly more PoTs related symptoms such as chest pain, dizziness, fainting spells (D1 and D2), palpitations, and shortness of breath (D1) compared to hEDS/HSD + IBS (*p* ≤ 0.05).

Patients with overlap hEDS/HSD + IBS had significantly higher median PHQ‐12 scores than hEDS/HSD alone (*p* <0.01). This cluster had significantly more fibromyalgia pain type symptoms such as back pain, pains in the arms/legs/joints and headaches versus hEDS/HSD alone (*p* ≤ 0.05) (D1 and D2). Additionally, in D2 these patients had significantly more dizziness, fainting spells, palpitations, shortness of breath, lethargy, and insomnia versus hEDS/HSD alone (*p* ≤ 0.05).

#### 3.3.2 Health related quality of life (Tables 5 and 6)

Health Related Quality of life (HrQoL) was lowest in the hEDS/HSD + FFD + IBS cluster in all eight SF‐8/SF‐36 domains (aside from mental health in D2) compared to hEDS/HSD alone (*p* ≤ 0.05). The hEDS/HSD + FFD + IBS cluster in D1 also had a worse HrQol compared to hEDS/HSD + IBS in the physical functioning, physical health, bodily pain, general health, social functioning, and emotional domains (*p* ≤ 0.05). In general, HrQoL in hEDS/HSD + IBS was lower than hEDS/HSD alone but higher than hEDS/HSD + FFD + IBS, however, this trend was not constantly statistically significant between datasets in all domains as demonstrated in Tables 5 and 6.

#### 3.3.3 Depression and anxiety (Tables 5 and 6)

Both the hEDS/HSD + FFD + IBS (*p* <0.0001) and hEDS/HSD + IBS (*p* <0.05) clusters had significantly higher depression scores than hEDS/HSD alone however, depression scores were highest in the hEDS/HSD + FFD + IBS cluster. Additionally, the hEDS/HSD + FFD + IBS group had significantly higher anxiety scores than both hEDS/HSD + IBS (*p* ≤ 0.05) and hEDS/HSD alone (*p* < 0.0001). No differences in anxiety scores were seen between hEDS/HSD + IBS versus hEDS/HSD alone (D1 and D2).

#### 3.3.4 PoTS status (D1) and autonomic symptoms via COMPASS questionnaire (D2) (Tables 5 and 6)

PoTS status and autonomic symptoms were highest in the hEDS/HSD + FFD + IBS cluster. Specifically, In D1, patients in this cluster were more likely to have co‐morbid PoTS than both the hEDS/HSD alone (*p* = 0.033) and hEDS/HSD + IBS clusters (*p* = 0.043). There was no significant difference in PoTS symptoms between hEDS/HSD + IBS versus hEDS/HSD alone.

PoTs related symptoms (assessed via COMPASS questionnaire) in D2 were also highest in the hEDS/HSD + FFD + IBS cluster in all domains compared to hEDS/HSD alone (*p* ≤ 0.01). The highest compass scores in the hEDS/HSD + FFD + IBS cluster were present in the orthostatic domain (vs: hEDS/HSD alone, *p* < 0.0001; hEDS/HSD + IBS, *p* = 0.003), followed by the GI domains, diarrhea (vs: hEDS/HSD alone, *p* <0.0001; hEDS/HSD + IBS, *p* = 0.023) and dyspepsia (vs: hEDS/HSD alone, *p* < 0.0001; hEDS/HSD + IBS, *p* < 0.0001).

The hEDS/HSD + IBS cluster had more orthostatic (*p* < 0.0001), GI (*p* < 0.0001) and urinary symptoms (*p* < 0.0001) compared to hEDS/HSD alone.

### 3.4 Beighton score (D2) (Table 6)

There were no significant differences in mean Beighton score between any of the three clusters.

## 4 DISCUSSION

Our study demonstrates that two main DGBI clusters exist in a hEDS/HSD cohort: those with mainly functional foregut disorders (functional dyspepsia, dysphagia, functional heartburn, belching, chronic nausea and vomiting and rumination) with overlapping IBS (hEDS/HSD + FFD + IBS; Cluster 0) and, a cluster with predominantly IBS (hEDS/HSD + IBS; Cluster 1).

The participants in Cluster 2 reflect those without DGBI symptoms. This may be likely to several factors. First off, variability in symptom expression means that hEDS/HSD has a wide range of clinical presentations. While there is a known association between hEDS/HSD and gastrointestinal issues, not all individuals with hEDS/HSD experience these symptoms. This variability may be due to differences in genetic, environmental, and physiological factors that influence how hEDS/HSD manifests in each individual, though this is yet to be investigated. Furthermore, although hEDS/HSD is associated with disorders of gut‐brain interaction (DGBI), the link is not universal. The development of DGBI symptoms may depend on additional factors such as diet, lifestyle, stress levels, coexisting medical conditions, or even differences in gut microbiota. Unfortunately, we cannot determine these additional factors from our current study, but this would prove to be a valuable study for the future.

A key advantage of the clustering approach used to identify these clusters is the reduced risk of bias. The algorithm used, autonomously identifies and produces clusters based on underlying patterns in the data, rather than relying on pre‐determined assumptions. Consequently, the use of this methodology removes elements of human bias and allows for a more objective analysis of the relationships between hEDS/HSD and DGBIs.

We demonstrate that the clusters with DGBI overlap have significantly more somatic symptoms, health related impairment, autonomic symptoms, and psychopathology compared to those with hEDS/HSD alone. Additionally, we demonstrate that anxiety disorder, autonomic symptoms and PoTS are considerably increased in those with hEDS/HSD and predominantly foregut disorders (hEDS/HSD + FFD + IBS) when compared to both hEDS/HSD + IBS and hEDS/HSD alone; this is a novel finding and has not been previously demonstrated in the literature.

Our findings that IBS and FD present as the two most common DGBI in hEDS/HSD corroborate with published literature which shows a prevalence of 23%–48% of IBS in hEDS/HSD^4^, ^21^, ^22^ and inversely, a 41.9% prevalence of hEDS/HSD in IBS^23^; A 28%–68% prevalence of FD in hEDS/HSD,^21^, ^24^ and inversely a 55% prevalence of hEDS/HSD in FD.^25^

Additionally, similar to our current study where we demonstrate an overlap of 67.6% between IBS and FD in hEDS/HSD (Cluster 0), previous studies have also shown a 68% overlap between IBS and FD in patients attending tertiary care GI clinics.^26^ Patients with overlap IBS/FD experience poorer quality of life and more severe clinical manifestations of disease compared to either IBS or FD alone.^26^ We demonstrate that this is also the case within our hEDS/HSD cohort, whereby patients with overlapping hEDS/HSD + FFD + IBS present with a more severe clinical phenotype, with a poorer quality of life, increased somatization, dysautonomia, PoTS and an increased psychological burden when compared to hEDS/HSD + IBS and hEDS/HSD alone. This is a unique finding as existing research on hEDS/HSD has primarily categorized patient groups into hEDS/HSD + DGBI in a general sense, without further differentiating the impact of specific overlapping DGBI's on the clinical presentation.

Our study raised a number of clinically significant themes that warrant further explanation, particularly as neurogastroenterology and gastroenterology clinics are progressively seeing an increase in the number of patients with hEDS/HSD.^3^, ^5^, ^27^ We demonstrated that overall, non‐GI related somatization in hEDS/HSD is greater in those with IBS, and further amplified by the additional presence of FFD. Based on previous studies one could hypothesize that this amplification may be due to the process of viscero‐somatic hypersensitivity^28^ due to central sensitisation of spinal dorsal horn neurons where visceral and somatic afferents converge, due to repetitive nociceptive involvement resulting from recurring injury^29^, ^30^ to visceral or somatic tissues. However, this may not fully explain the somatization seen in these patients as viscerosomatic convergence usually occurs over the somatic areas of the abdomen such as the skin and muscles of the abdominal wall.^31^, ^32^ Therefore, the occurrence of widespread pain and fibromyalgia‐like symptoms which we demonstrate in our study cannot be explained by viscero‐somatic hypersensitivity alone. Another plausible explanation for these symptoms may be the heightened psychological comorbidity and hypervigilance that is commonly observed in patients with DGBI.^33^, ^34^ Studies have shown that patients with psychological comorbidity may exhibit reduced descending pain pathway inhibition of the spinal dorsal horn neurons.^35^ Consequently, in patients with DGBI and associated psychological morbidity, it is possible that the descending pain inhibition pathway is less effective in suppressing incoming nociceptive signals hence leading to widespread pain including back pain and pain in the limbs. This hypothesis should be tested in future studies.

Dysregulation of the autonomic nervous system (ANS) in patients with overlap DGBI and hEDS/HSD is described in the form of parasympathetic withdrawal. Kolacz et al. demonstrated that patients with overlap hEDS/HSD and functional abdominal pain disorders (FAPD) have lower vagal efficiency, and suboptimal heart periods and ventral vagal parasympathetic tone when supine, compared to those with FAPD without hEDS/HSD and healthy controls.^36^ We demonstrated an increase in PoTS in the FFD + IBS cluster and generally higher COMPASS questionnaire based autonomic scores and symptoms compared to hEDS/HSD alone and the overlap hEDS/HSD + IBS clusters. This finding may have two possible explanations. Firstly, autonomic nervous system dysfunction with sympathetic nervous activation and parasympathetic nervous system withdrawal has been described in functional dyspepsia^37^ and therefore this may be a primary problem of functional dyspepsia rather than hEDS/HSD; or inversely, it may be that autonomic symptoms in the hEDS/HSD + FFD + IBS cluster result in gut disorders such as functional dyspepsia. Previous studies have demonstrated that the vagus nerve has a greater innervation for the foregut versus hindgut^38^ and therefore parasympathetic nervous system dysautonomia is more likely to be associated with FFD symptoms such as functional dyspepsia compared to IBS. Furthermore, FD and other FFD are more common than lower GI symptoms in patients with PoTS overlap versus no overlap.^22^ Studies have also demonstrated that in PoTS there is increased sympathetic activity and reduced parasympathetic activity.^36^ Whilst the sympathetic nervous system is stimulatory for the heart, its splanchnic division is inhibitory for gut motor function while spinal visceral afferents are involved in nociception. This pattern of dysautonomia may contribute to the development of delayed gastric emptying and visceral hypersensitivity, which are observed in functional dyspepsia.

There are existing reports of ANS dysfunction in the functional foregut disorders functional dyspepsia, chronic unexplained nausea and vomiting, gastroesophageal reflux disease and in IBS.^37^, ^39^, ^40^, ^41^ Plausibly, our hEDS/HSD + FFD + IBS cluster is picking up a group of individuals who have autonomic dysfunction as the underlying pathophysiology. Therefore, treatment of PoTS and dysautonomia could have therapeutic potential for FFD and IBS which should be explored in future studies. A potential therapeutic target that warrants investigation for this overlapping cohort is pyridostigmine.

Pyridostigmine, a cholinesterase antagonist, has been reported to increase gastrointestinal motility resulting in improvement in symptoms of chronic constipation^42^ as well as increasing gastric contractions in autoimmune gastrointestinal dysmotility.^43^, ^44^ Administration of pyridostigmine has also been associated with a reduction in orthostatic tachycardia symptoms^45^, ^46^; consequently, this could be explored in those with overlap hEDS/HSD + FFD + IBS and associated ANS dysfunction. This hypothesis should be rigorously tested in future clinical trials.

Evaluation of psychopathology in our study demonstrated that only those with FFD and IBS overlap had an increase in anxiety symptoms when compared to hEDS/HSD alone. It is interesting that this group also experienced PoTS/autonomic symptoms. As described above, patients with PoTS can have increased sympathetic activity and parasympathetic withdrawal^36^; a pattern of autonomic dysfunction also associated with anxiety disorders.^47^ Thus, it is feasible that anxiety in patients with hEDS/HSD + FFD + IBS is mediated by the dysautonomia due to a labile heart rate response to physical or psychological stressors, although the higher symptom burden compared to other sub‐groups may also be a contributory factor.

Our data demonstrates that depression scores in hEDS/HSD were significantly increased in IBS patients, and further amplified by the presence of both IBS and FFD. Previous studies have demonstrated that those with overlap of IBS and functional dyspepsia presenting to gastroenterology clinics have a greater prevalence of depression than the non‐overlap patients.^48^ This may be a consequence of increased symptom burden and poorer quality of life in these comorbid patients^48^ which may explain why in our study, the hEDS/HSD patients with IBS and FFD overlap present as the most depressed group.

Finally, quality of life was decreased in hEDS/HSD with overlap IBS and further decreased in the presence of FFD in both datasets. The deterioration of quality of life in these groups could be explained by the increasing co‐morbidity including somatization, psychopathology and dysautonomia in the hEDS/HSD + IBS + FFD group.

The study does have its limitations, with the first pertaining to the use of retrospective datasets which may have led to the exclusion of valuable information. Furthermore, the collection of data from two different study cohorts, dataset 2 which used the ROME III criteria for a diagnosis of IBS whilst dataset 1 used the more stringent ROME IV, may have resulted in both selection and response bias. Additionally, upon statistical analysis, differences in sex, age and more specifically, DGBI prevalence were observed between the two datasets. This may have been due to dataset 1's origin from a patient support organization, which may inherently select for a more symptomatic population. We aimed to determine the extent of the mentioned issues by applying a logistic regression analysis prior to clustering to ensure that the study dataset used did not con‐found our clustering results. Our regression analysis demonstrated that when DGBI and hEDS/HSD diagnoses were mixed from both cohorts, the regression model was unable to determine which cohort patients came from based upon their DGBI and hEDS/HSD diagnosis. However, nevertheless, the differences between the two separate cohorts once again became apparent upon downstream statistical analysis, hence demonstrating differences in patient profiles in those presenting to clinics and attending patient support groups. Future studies should look to only recruit from one source to avoid discrepancies between datasets. Another limitation arises from utilizing questionnaires in studies as patients may unconsciously or consciously inflate their symptoms on a questionnaire. However, this limitation is applicable to all datasets involving questionnaires and is a phenomenon which may also manifest in face‐to‐face consultations.^49^ Lastly, a validated method for assessing autonomic symptoms/PoTs such as the tilt table test would be helpful to draw more valuable conclusions.

Despite these limitations, this study demonstrates that within the wider hEDS/HSD population two main clusters with coexisting DGBI exist. Those with predominantly IBS who have high somatization, depression, poor quality of life and those with predominantly foregut disorders with a high prevalence of overlap DGBIs, and significantly more anxiety, PoTs, autonomic symptoms, somatization and worse quality of life. It can be speculated that treatment of PoTS in the FFD + IBS cluster may have a beneficial effect on some of the foregut and IBS symptoms. Furthermore, hEDS/HSD groups with overlapping DGBI would benefit from a multidisciplinary approach with input from healthcare professionals such as psychiatrists, pain management specialists as well as cardiologists and neurologists. Currently, there is no multidisciplinary commissioned service available for such patients and this requires urgent consideration by healthcare commissioners. Finally, our study provides ideas for future research to explore the pathophysiological factors that link DGBI and hEDS/HSD such as altered descending pain inhibitory pathways, dysautonomia and benefits of multidisciplinary approach to treatment which have the potential to improve the management of these complex patients.

## AUTHOR CONTRIBUTIONS

QA conceived the study concept. AC, AF, JKR and QA conceived the study design. AF, IA, and OP collected the study data. JKR and KT trained AC to conduct analysis of data. AC analyzed the data and wrote the manuscript. All authors edited the manuscript and approved the final version.

## CONFLICT OF INTEREST STATEMENT

Anisa Choudhary—No conflict of interest. Asma Fikree—No conflict of interest. James K. Ruffle—No conflict of interest. Kazuya Takahashi—No conflict of interest. Olafur S. Palsson—No conflict of interest. Imran Aziz—No conflict of interest. Qasim Aziz—Takeda pharmaceuticals: received funding for commercial clinical trial, Clasado BioSciences: received funding for commercial clinical trial, Falk Pharma: received funding for commercial clinical trial, Bromatech: honorarium for lecture.

## Supporting information


Appendix S1.

Appendix S2.

Appendix S3.

Appendix S4.

## Data Availability

Research data are not shared.
