# Supplementary material for: A machine learning approach to stratify patients with hypermobile Ehlers‐Danlos syndrome/hypermobility spectrum disorders according to disorders of gut brain interaction, comorbidities and quality of life
Source: Neurogastroenterol Motil. 2024 Nov 14;37(1):e14957. doi: 10.1111/nmo.14957 (PMC11650402; doi:10.1111/nmo.14957)
Supplement: Supplementary file 1 — Appendix S1. [file NMO-37-e14957-s001.pdf]

Supplementary Table 1. Demonstrating the demographics and proportion of patients from primary, secondary and tertiary care in dataset 2 (*n* = 379).[1]

**Clinical Setting (*n*)**	**Age Range (years)**	**Sex (*n*)**
Primary Care (24)	18 – 69	Female (16)<br>Male (8)
Secondary Care (180)	19 - 70	Female (123)<br>Male (57)
Tertiary Care (122)	19-70	Female (104)<br>Male (18)

[1] Clinical setting was not stated for 53 of the patients included in dataset 2.

Supplementary Table 2. Demonstrating the proportion of DGBIs in primary, secondary and tertiary care in dataset 2.[1]

**DGBI**	**Primary Care** *n* = 24 n, (%)	**Secondary Care** *n* = 180 n, (%)	**Tertiary Care** *n* = 122 n, (%)
IBS	0	49 (27.2)	74 (60.7)
Functional Dyspepsia	0	44 (24.4)	68 (55.7)
Dysphagia	0	8 (4.4)	15 (12.3)
Proctalgia Fugax	0	12 (6.7)	16 (13.1)
Rumination	0	0	0
Functional Heartburn	0	1 (0.6)	2 (1.6)
Faecal Incontinence	0	0	1 (0.8)
Chronic Nausea & Vomiting	0	4 (2.2)	2 (1.6)
Functional Chest pain	0	8 (4.4)	7 (5.7)
Functional Constipation	0	2 (1.1)	3 (2.5)
Belching	0	0	1 (0.8)
Cyclic Vomiting	0	0	0
Unspecified functional bowel disorder	0	7 (3.9)	1 (0.8)

Functional bloating	0	0	0
Globus	0	3 (1.7)	9 (7.4)
Central Abdominal Pain Syndrome	0	2 (1.1)	1 (0.8)
Functional Diarrhoea	0	44 (24.4)	4 (3.3)

Supplementary Table 3. Demonstrating sex and age distribution in dataset 1 versus dataset 2

**Demographics**	**Dataset 1 (n = 665)**	**Dataset 2 (n = 379)**	**P value**
Sex (n)	Female (641)<br>Male (24)	Female (276)<br>Male (103)	< 0.001
Age (mean)	39 years	41 years	0.021

Supplementary Table 4. Demonstrating the prevalence of DGBIs in dataset 1 versus dataset 2

**DGBI**	**Dataset 1 Prevalence (n = 665)**<br><br>***N (%)***	**Dataset 2 Prevalence (*n*= 379)**<br><br>***N (%)***	**P value**
Irritable Bowel Syndrome	371 (55.8)	124 (32.7)	< 0.001
Functional Heartburn	123 (18.5)	3 (0.8)	<0.001
Functional chest pain	84 (12.6)	16 (4.2)	<0.001
Globus	11 (1.7)	12 (3.2)	0.109
Dysphagia	282 (42.4)	24 (6.3)	< 0.001
Functional dyspepsia	382 (57.4)	113 (29.8)	< 0.001
Belching	77 (11.6)	2 (0.5)	< 0.001
Rumination	198 (29.8)	0	< 0.001
Cyclical Vomiting Syndrome	102 (15.3)	6 (1.6)	< 0.001

Cyclical Vomiting	66 (9.9)	0	< 0.001
Functional Constipation	81 (12.2)	5 (1.3)	< 0.001
Functional Diarrhoea	34 (5.1)	10 (2.6)	0.056
Functional Bloating	18 (2.7)	0	0.001
Unspecified Functional Bowel Disorder	62 (9.3)	8 (2.1)	<0.001
Central Abdominal Pain Syndrome	3 (0.5)	3 (0.8)	0.484
Faecal Incontinence	118 (17.7)	1 (0.3)	< 0.001
Proctalgia Fugax	195 (29.3)	29 (7.7)	< 0.001

Supplementary Table 5. Demonstrating the demographics of the 61 patients not clustered and classified as ‘noise.’

**Age Range (years)**	**Sex (*n*)**
20 - 67	Female (55)<br>Male (6)

Supplementary Table 6. Demonstrating DGBI’s in the 61 patients not clustered and classified as ‘noise.’

**DGBI**	n, (%)
IBS	0
Functional Dyspepsia	1 (1.6)
Dysphagia	9 (14.8)
Proctalgia Fugax	11 (18)
Rumination	10 (16.4)
Functional Heartburn	4 (6.6)
Faecal Incontinence	4 (6.6)
Chronic Nausea & Vomiting	1 (1.6)
Functional Chest pain	4 (6.6)
Functional Constipation	0
Belching	3 (4.9)
Cyclic Vomiting	0
Unspecified functional bowel disorder	61 (100)
Functional bloating	0
Globus	1 (1.6)
Central Abdominal Pain Syndrome	1 (1.6)
Functional Diarrhoea	0
