# Supplementary material for: A machine learning approach to stratify patients with hypermobile Ehlers‐Danlos syndrome/hypermobility spectrum disorders according to disorders of gut brain interaction, comorbidities and quality of life
Source: Neurogastroenterol Motil. 2024 Nov 14;37(1):e14957. doi: 10.1111/nmo.14957 (PMC11650402; doi:10.1111/nmo.14957)
Supplement: Supplementary file 2 — Appendix S2. [file NMO-37-e14957-s003.pdf]

Supplementary Document 1. Background and interpretation of the questionnaires used within the study

Patient Health Questionnaire-12 (PHQ-12)

The PHQ-12 is the adjusted version of the PHQ-15 Questionnaire which aims to assess the presence and severity of somatic symptoms over the preceding seven days. The PHQ-12 excludes questions relating to GI symptoms and provides information on non-GI somatisation. It encompasses 6-items evaluating pain in the back, limbs, head, chest, menstrual pain and dyspareunia; 4-items evaluating autonomic symptoms including dizziness, fainting spells, palpitations, and shortness of breath; 1-item for fatigue and lastly, 1-item for insomnia. The questionnaire is scored out of 24 with scores of ≤ 3 representing mild somatisation or no somatisation, 4-7 low somatisation, 8-12 medium somatisation and ≥ 13 indicating high somatisation.[10,12]

Short Form Survey (SF-8) and (SF-36) questionnaires

The short form surveys aim to assess quality of life. The SF-8 contains 8 items which assess quality of life over the preceding four weeks. The domains included are general health, physical functioning, the role of poor physical health limiting daily activities, bodily pain, vitality, social activities, mental health and the role of mental health limiting daily activities.[13] The SF-36 evaluates the matching domains as the SF-8, however uses 36 questions to obtain total scores for each of the eight domains.[16]

General anxiety disorder questionnaire (GAD-7)

The GAD-7 is a 7-item questionnaire assessing the presence and severity of generalized anxiety over the past two weeks. Scores are calculated out of 21, with sores of 0-4 establishing minimal anxiety, 5-9 mild anxiety, 10 – 14 moderate anxiety and 15 – 21 severe anxiety.[14]

Patient health questionnaire-9 (PHQ-9)

The PHQ-9 is a 9-item questionnaire assessing the presence and severity of depression over the preceding two weeks. Scores are calculated over of 27 with 1-4 indicating minimal depression, 5-9 mild depression, 10-14 moderate depression, 15-19 moderately severe depression and 20-27 severe depression.[15]

The Symptom Checklist -90 (SCL-90)

The SCL-90 is a 90-item questionnaire assessing the presence of a range of psychological symptoms including both anxiety and depression. Results are scored from 0 – 4, with the higher the score, the more severe the symptoms.[17]

COMPASS Questionnaire

The COMPASS questionnaire is a 31-item questionnaire which assesses the presence, frequency and severity of autonomic symptoms. The questionnaire is centered on the 169 question Autonomic Symptom Profile (ASP) questionnaire. The questionnaire evaluates 6 domains including orthostatic intolerance and vasomotor, secretomotor, gastrointestinal, bladder and pupillomotor symptoms. The questionnaire is scored out of 75 with a higher result indicating greater autonomic dysfunction.[18]

10. Lam CY, Palsson OS, Whitehead WE, et al. Rome IV Functional Gastrointestinal Disorders and Health Impairment in Subjects With Hypermobility Spectrum Disorders or Hypermobile Ehlers-Danlos Syndrome. Clin Gastroenterol Hepatol 2021;19:277-287 e3.
12. Spiller RC, Humes DJ, Campbell E, et al. The Patient Health Questionnaire 12 Somatic Symptom scale as a predictor of symptom severity and consulting behaviour in patients with irritable bowel syndrome and symptomatic diverticular disease. Aliment Pharmacol Ther 2010;32:811-20.
13. Ware JE, Kosinski M, Dewey JE, et al. How to score and interpret single-item health status measures: a manual for users of the SF-8 health survey. Lincoln, RI: QualityMetric Incorporated 2001;15:5.
14. Spitzer RL, Kroenke K, Williams JB, et al. A brief measure for assessing generalized anxiety disorder: the GAD-7. Arch Intern Med 2006;166:1092-7.
15. Kroenke K, Spitzer RL, Williams JB. The PHQ-9: validity of a brief depression severity measure. J Gen Intern Med 2001;16:606-13.
16. McHorney CA, Ware JE, Jr., Raczek AE. The MOS 36-Item Short-Form Health Survey (SF-36): II. Psychometric and clinical tests of validity in measuring physical and mental health constructs. Med Care 1993;31:247-63.
17. Derogatis LR, Rickels K, Rock AF. The SCL-90 and the MMPI: a step in the validation of a new self-report scale. Br J Psychiatry 1976;128:280-9.
18. Sletten DM, Suarez GA, Low PA, et al. COMPASS 31: a refined and abbreviated Composite Autonomic Symptom Score, In Mayo Clinic Proceedings, Elsevier, 2012.
