# Supplementary material for: A machine learning approach to stratify patients with hypermobile Ehlers‐Danlos syndrome/hypermobility spectrum disorders according to disorders of gut brain interaction, comorbidities and quality of life
Source: Neurogastroenterol Motil. 2024 Nov 14;37(1):e14957. doi: 10.1111/nmo.14957 (PMC11650402; doi:10.1111/nmo.14957)
Supplement: Supplementary file 3 — Appendix S3. [file NMO-37-e14957-s002.docx]

Supplementary Table 1. Demonstrating the demographics and proportion of patients from primary, secondary and tertiary care in dataset 2 (*n* = 379).^[[1]](#footnote-1)^

| **Clinical Setting (*n*)** | **Age Range (years)** | **Sex (*n*)** |
| --- | --- | --- |
| Primary Care (24) | 18 – 69 | Female (16)  Male (8) |
| Secondary Care (180) | 19 - 70 | Female (123)  Male (57) |
| Tertiary Care (122) | 19-70 | Female (104)  Male (18) |

Supplementary Table 2. Demonstrating the proportion of DGBIs in primary, secondary and tertiary care in dataset 2.^1^

| **DGBI** | **Primary Care**  *n* = 24  n, (%) | **Secondary Care**  *n* = 180  n, (%) | **Tertiary Care**  *n* = 122  n, (%) |
| --- | --- | --- | --- |
| IBS | 0 | 49 (27.2) | 74 (60.7) |
| Functional Dyspepsia | 0 | 44 (24.4) | 68 (55.7) |
| Dysphagia | 0 | 8 (4.4) | 15 (12.3) |
| Proctalgia Fugax | 0 | 12 (6.7) | 16 (13.1) |
| Rumination | 0 | 0 | 0 |
| Functional Heartburn | 0 | 1 (0.6) | 2 (1.6) |
| Faecal Incontinence | 0 | 0 | 1 (0.8) |
| Chronic Nausea & Vomiting | 0 | 4 (2.2) | 2 (1.6) |
| Functional Chest pain | 0 | 8 (4.4) | 7 (5.7) |
| Functional Constipation | 0 | 2 (1.1) | 3 (2.5) |
| Belching | 0 | 0 | 1 (0.8) |
| Cyclic Vomiting | 0 | 0 | 0 |
| Unspecified functional bowel disorder | 0 | 7 (3.9) | 1 (0.8) |
| Functional bloating | 0 | 0 | 0 |
| Globus | 0 | 3 (1.7) | 9 (7.4) |
| Central Abdominal Pain Syndrome | 0 | 2 (1.1) | 1 (0.8) |
| Functional Diarrhoea | 0 | 44 (24.4) | 4 (3.3) |
|  |  |  |  |

Supplementary Table 3. Demonstrating sex and age distribution in dataset 1 versus dataset 2

| **Demographics** | **Dataset 1**  **(n = 665)** | **Dataset 2**  **(n = 379)** | **P value** |
| --- | --- | --- | --- |
| Sex (n) | Female (641)  Male (24) | Female (276)  Male (103) | < 0.001 |
| Age (mean) | 39 years | 41 years | 0.021 |

Supplementary Table 4. Demonstrating the prevalence of DGBIs in dataset 1 versus dataset 2

| **DGBI** | **Dataset 1 Prevalence**  **(n = 665)**  ***N (%)*** | **Dataset 2 Prevalence**  **(*n*= 379)**  ***N (%)*** | **P value** |
| --- | --- | --- | --- |
| Irritable Bowel Syndrome | 371 (55.8) | 124 (32.7) | < 0.001 |
| Functional Heartburn | 123 (18.5) | 3 (0.8) | <0.001 |
| Functional chest pain | 84 (12.6) | 16 (4.2) | <0.001 |
| Globus | 11 (1.7) | 12 (3.2) | 0.109 |
| Dysphagia | 282 (42.4) | 24 (6.3) | < 0.001 |
| Functional dyspepsia | 382 (57.4) | 113 (29.8) | < 0.001 |
| Belching | 77 (11.6) | 2 (0.5) | < 0.001 |
| Rumination | 198 (29.8) | 0 | < 0.001 |
| Cyclical Vomiting Syndrome | 102 (15.3) | 6 (1.6) | < 0.001 |
| Cyclical Vomiting | 66 (9.9) | 0 | < 0.001 |
| Functional Constipation | 81 (12.2) | 5 (1.3) | < 0.001 |
| Functional Diarrhoea | 34 (5.1) | 10 (2.6) | 0.056 |
| Functional Bloating | 18 (2.7) | 0 | 0.001 |
| Unspecified Functional Bowel Disorder | 62 (9.3) | 8 (2.1) | <0.001 |
| Central Abdominal Pain Syndrome | 3 (0.5) | 3 (0.8) | 0.484 |
| Faecal Incontinence | 118 (17.7) | 1 (0.3) | < 0.001 |
| Proctalgia Fugax | 195 (29.3) | 29 (7.7) | < 0.001 |

Supplementary Table 5. Demonstrating the demographics of the 61 patients not clustered and classified as ‘noise.’

| **Age Range (years)** | **Sex (*n*)** |
| --- | --- |
| 20 - 67 | Female (55)  Male (6) |

Supplementary Table 6. Demonstrating DGBI’s in the 61 patients not clustered and classified as ‘noise.’

| **DGBI** | n, (%) |
| --- | --- |
| IBS | 0 |
| Functional Dyspepsia | 1 (1.6) |
| Dysphagia | 9 (14.8) |
| Proctalgia Fugax | 11 (18) |
| Rumination | 10 (16.4) |
| Functional Heartburn | 4 (6.6) |
| Faecal Incontinence | 4 (6.6) |
| Chronic Nausea & Vomiting | 1 (1.6) |
| Functional Chest pain | 4 (6.6) |
| Functional Constipation | 0 |
| Belching | 3 (4.9) |
| Cyclic Vomiting | 0 |
| Unspecified functional bowel disorder | 61 (100) |
| Functional bloating | 0 |
| Globus | 1 (1.6) |
| Central Abdominal Pain Syndrome | 1 (1.6) |
| Functional Diarrhoea | 0 |
|  |  |

1. Clinical setting was not stated for 53 of the patients included in dataset 2. [↑](#footnote-ref-1)
